# A novel endoscopic approach for efficient and safe resection of tumors at the appendiceal orifice

**DOI:** 10.1055/a-2609-7060

**Published:** 2025-06-18

**Authors:** Shuhei Fukunaga, Shinpei Minami, Tomonori Cho, Daiki Ohzono, Hiroshi Tanaka, Tomoyuki Nakane, Michita Mukasa

**Affiliations:** 1 12904 Division of Gastroenterology, Department of Medicine, Kurume University School of Medicine, Kurume, Fukuoka, Japan

Endoscopic resection of colorectal tumors at the appendiceal orifice remains challenging due to its anatomical complexity. Conventional endoscopic mucosal resection (EMR) often fails to achieve en-bloc resection, while endoscopic submucosal dissection (ESD) carries a high risk of perforation. Although full-thickness resection is effective, it is not always available or necessary. We report a cap-assisted endoscopic mucosal resection with an over-the-scope clip (EMRO-C) technique, facilitating efficient en-bloc resection at the appendiceal orifice ( Video 1 ). A 70-year-old male patient had a Paris type 0-IIa, 20-mm tumor at the appendiceal orifice ( Fig. 1 **a** ). Initially, a PCF-H290 endoscope (Olympus), equipped with an over-the-scope (OTS) clip system (Ovesco Endoscopy GmbH, Tübingen) was utilized ( Fig. 1 **b** ). The clip was deployed directly beneath the lesion, creating a pseudo-polypoid elevation ( Fig. 1 **c** ). The endoscope was then withdrawn, and the MAJ-290 distal attachment (Olympus) was placed. The SD221-L25 snare (Olympus) was positioned ( Fig. 1 **d** ). The elevated lesion was completely resected using full suction into the attachment ( Fig. 1 **e** ). Endoscopic resection was successfully performed, and the OTS clip effectively prevented perforation ( Fig. 1 **f** ). No adverse events, including perforation or bleeding, occurred during or after the procedure. Histopathological examination confirmed tubular adenoma, with negative margins ( Fig. 2 **a, b** ). EMR using the OTS clip technique, termed EMRO, has been reported as a promising method for treating duodenal neuroendocrine tumors 1 . Despite its high efficacy, achieving successful snaring can be challenging. We previously reported that EMRO-C is useful for lesions that are difficult to resect using a snare 2 . Our findings highlight that EMRO-C is also an efficient technique for en-bloc resection of colorectal tumors at the appendiceal orifice.

**Fig. 1:**
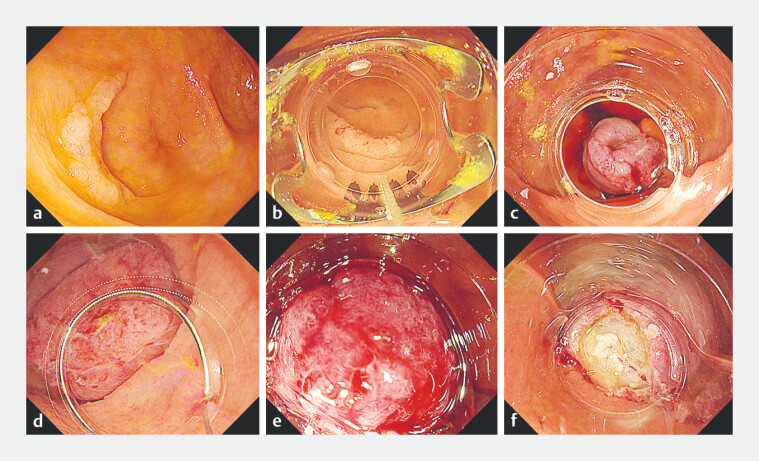
Endoscopic images of the EMRO-C procedure at the appendiceal orifice. **a** A Paris type 0-IIa tumor at the appendiceal orifice. **b** Initial endoscopic view of the lesion with the OTS clip system. **c** OTS clip deployed beneath the lesion. **d** The snare is positioned with a distal attachment on the endoscope. **e** Complete resection of the lesion using suction. **f** Image after resection. Abbreviation: EMRO-C, endoscopic mucosal resection with an over-the-scope clip; OTS, over-the-scope.

**Fig. 2:**
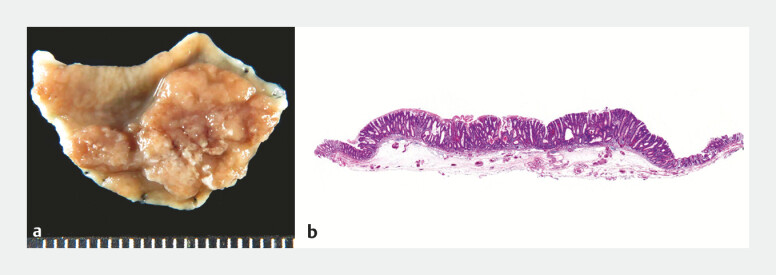
Histopathological images. **a** Resected specimen. **b** Hematoxylin-eosin staining, low magnification.

Step-by-step demonstration of the EMRO-C technique for colorectal tumor resection at the appendiceal orifice.Video 1

Endoscopy_UCTN_Code_TTT_1AQ_2AD_3AC

## Notes

Endoscopy E-Videos https://eref.thieme.de/e-videos
*E-Videos* is an open access online section of the journal *Endoscopy* , reporting on interesting cases and new techniques in gastroenterological endoscopy. All papers include a high-quality video and are published with a Creative Commons CC-BY license. Endoscopy E-Videos qualify for HINARI discounts and waivers and eligibility is automatically checked during the submission process. We grant 100% waivers to articles whose corresponding authors are based in Group A countries and 50% waivers to those who are based in Group B countries as classified by Research4Life (see: https://www.research4life.org/access/eligibility/ ). This section has its own submission website at https://mc.manuscriptcentral.com/e-videos .
